# The role of co-infection in the pathogenesis of acute SARS-CoV-2 infection and development of post-acute sequelae: A perspective

**DOI:** 10.7554/eLife.106308

**Published:** 2025-11-17

**Authors:** Timothy J Henrich, Christopher P Montgomery, Joerg Graf, Nahed Ismail, Sindhu Mohandas, Mehul S Suthar, Hassan Brim, John M Coffin, Aayush Pagaria, Jeisac Guzmán Rivera, Urmila Vudali, Paul Keim, Guangming Zhong, Rebecca McGrath, Belinda Edwards, Adolfo García-Sastre, Maria Laura Gennaro

**Affiliations:** 1 Division of Experimental Medicine, University of California, San Francisco San Francisco United States; 2 Center for Microbial Pathogenesis, Abigail Wexner Research Institute; Division of Critical Care Medicine, Nationwide Children’s Hospital Columbus United States; 3 Department of Pediatrics, The Ohio State University College of Medicine Columbus United States; 4 Pacific Biosciences Research Center, University of Hawaiʻi at Mānoa Honolulu United States; 5 Department of Pathology, University of Illinois at Chicago Chicago United States; 6 Division of Infectious Diseases, Children’s Hospital Los Angeles, Keck School of Medicine, University of Southern California Los Angeles United States; 7 Emory Vaccine Center, Department of Pediatrics, Emory University School of Medicine Atlanta United States; 8 Howard University Pathology Department Washington DC United States; 9 Department of Molecular Biology and Microbiology, Tufts University Boston United States; 10 PHRI, Rutgers New Jersey Medical School Newark United States; 11 Pathogen and Microbiome Institute, Northern Arizona University Flagstaff United States; 12 Department of Microbiology, Immunology and Molecular Genetics, University Texas Health San Antonio United States; 13 NIH RECOVER Research Initiative: Patient representative Attleboro United States; 14 NIH RECOVER Research Initiative: Patient representative Odenton United States; 15 Department of Microbiology, Global Health and Emerging Pathogens Institute, Department of Medicine, The Tisch Cancer Institute, The Icahn Genomics Institute, and Department of Pathology, Molecular and Cell-Based Medicine, Icahn School of Medicine at Mount Sinai New York United States; 16 Department of Medicine, Rutgers New Jersey Medical School Newark United States; University of the Witwatersrand South Africa; University of the Witwatersrand South Africa

**Keywords:** PASC, SARS-CoV-2, co-infection, long COVID

## Abstract

A major health challenge resulting from the COVID-19 pandemic is the manifestation of post-acute sequelae of SARS-CoV-2 (PASC). PASC (or long COVID) is a collective term used for clinical symptoms, various pathologies, and life-quality-changing functional impairment that persist for months to years after the initial SARS-CoV-2 infection. The mechanisms underlying PASC are not understood, although advances have been made in identifying factors that may contribute to long-term pathology. Recent data have emerged, showing an association between SARS-CoV-2 viral persistence and non-SARS-CoV-2 infections (pre-existing, viral reactivation, or new infections) in facilitating or mediating PASC. However, the heterogeneous nature and timing of co-infections have made it challenging to understand, interpret, and contextualize their contribution to PASC. Here, we summarize the impact of potential viral, bacterial, and fungal infections on SARS-CoV-2 pathogenesis, with a focus on their possible roles in the development of PASC. We also provide a framework to understand the mechanisms of PASC and inform basic, translational, and clinical research initiatives, including RECOVER, a large and ongoing research initiative to understand, treat, and prevent long COVID.

## Introduction

Infection with severe acute respiratory syndrome coronavirus-2 (SARS-CoV-2) may lead to persistent symptoms and sequelae that range from mild impairment of daily activities to severe long-term disability or death (Seeßle et al., 2022; Peluso et al., 2022c; Yang et al., 2022; Peluso and Deeks, 2022a; Mehandru and Merad, 2022). These symptoms, which relate to one or more organ systems and may be ongoing, relapsing, or new, define a multi-syndromic entity referred to as post-acute sequelae of SARS-CoV-2 infection (PASC). The World Health Organization describes PASC (or ‘long COVID’ or ‘post-COVID-19’) as a condition occurring in individuals with a history of probable or confirmed SARS-CoV-2 infection, 3 months from the onset of COVID-19, with symptoms that last for at least 2 months and cannot be explained by an alternative diagnosis (https://www.who.int/europe/news-room/fact-sheets/item/post-covid-19-condition). The US National Academies (National Academies of Sciences E, and Medicine et al., 2024) describe long COVID as a set of signs, symptoms, and conditions that continue or develop after initial infection, are present for 4 weeks or more, may be multisystemic, and present with relapsing-remitting patterns. Sequelae associated with other viral infections include the induction by infection of antibodies that enhance disease after subsequent reinfections, as is the case with dengue virus infections (Katzelnick et al., 2017). During the first months of the COVID-19 pandemic, the possibility of antibody-dependent enhancement (ADE) of disease associated with SARS-CoV-2 antibodies was a major concern for vaccine development. In fact, ADE has been documented for SARS-CoV-1 infections in laboratory and animal studies (Karthik et al., 2020). Nevertheless, there have been no documented cases of ADE in humans infected with SARS-CoV-2. The possibility that ADE might be involved in some of the PASC symptoms is therefore quite remote.

The worldwide burden of PASC is staggering: estimates of its incidence have ranged between 65 and 400 million by the end of 2023 (Davis et al., 2023). Although they incorporate risk reductions associated with roll-out of COVID-19 vaccines and reduced virulence of infection with newer SARS-CoV-2 strains (Al-Aly et al., 2022; Antonelli et al., 2022; Cortellini et al., 2023), these estimates may be conservative as they only represent cases arising from symptomatic infection (Davis et al., 2023). Given the burden, the potential effects of PASC on health, quality of life, and life expectancy are of concern worldwide. Finding causative or exacerbating mechanisms of PASC should be a public health priority.

PASC is of unknown etiology, presents heterogeneous clinical phenotypes, potentially impacts multiple organs, and is likely multifactorial. Affected organ systems include the neurological system, the gastrointestinal system, heart, lung, pancreas, and the reproductive system (Davis et al., 2023). Other organs, as well as blood vessels and the immune system, may also be affected (Davis et al., 2023; Altmann et al., 2023). While emerging data point to various major potential drivers of PASC (Peluso and Deeks, 2022a; Mehandru and Merad, 2022; Davis et al., 2023; Castanares-Zapatero et al., 2022), several causative hypotheses of PASC involve microbes or microbial products. For example, SARS-CoV-2 itself may persist in tissues after extrapulmonary viral dissemination during acute infection, resulting in lasting antigenic stimulus, inflammation, aberrant immune responses, and tissue damage (Altmann et al., 2023; Swank et al., 2023; Natarajan et al., 2022; Peluso et al., 2022b; Cheung et al., 2022; Acosta-Ampudia et al., 2022; Peluso et al., 2021b; Phetsouphanh et al., 2022). In addition to SARS-CoV-2, reactivation of latent viruses such as Epstein-Barr virus (EBV) and herpes simplex or herpes zoster viruses may be associated with PASC. Additionally, gut microbial dysbiosis and subsequent bacterial translocation to liver or other organs can lead to sustained inflammation, immune dysregulation, and tissue damage (Davis et al., 2023; Altmann et al., 2023; Peluso et al., 2023; Giron et al., 2022; Gold et al., 2021; Klein et al., 2023; Su et al., 2022; Grobbelaar et al., 2021; De Michele et al., 2022; Zuo et al., 2020). Moreover, it is well documented that co-infections exacerbate the severity of acute SARS-CoV-2 infection and worsen its outcome (Musuuza et al., 2021; Patton et al., 2023; Feldman and Anderson, 2021; Sreenath et al., 2021). Thus, it is tempting to consider the possibility that acute and chronic infectious diseases occurring prior to, during, or after acute SARS-CoV-2 infection may contribute to PASC development and phenotypic presentation. Understanding microbial contribution to PASC might dramatically improve our capabilities to diagnose and treat PASC, and to prevent it.

In this review, we outline how various viral, bacterial, and fungal co-infections may induce or facilitate the development of PASC. To do so, we use the current, limited evidence on the association between PASC and certain co-infections (mostly viral), the current knowledge on the impact of viral, bacterial, and fungal infections on the severity of acute SARS-CoV-2, and biological plausibility criteria derived from our collective understanding of the pathogenesis of various infectious diseases. While, as of this writing, all possible associations we discuss are within the realm of biological plausibility, they are, of necessity, speculative, and compelling evidence for a causal association of PASC with any microbial infection remains to be obtained. Here, our goal is to present the current state of understanding of the candidate microorganisms in the context of SARS-CoV-2 infection in the hope of stimulating and informing future research into this still mysterious and often debilitating condition. While changes in the composition of commensal microbiota may also be related to PASC, the potential role of dysbiosis in PASC has already been discussed in many reviews (e.g. Raj et al., 2024; Guo et al., 2023; Norouzi Masir and Shirvaliloo, 2022), requires specific considerations and is not included in the present review.

## Pandemic-associated surge of non-SARS-CoV-2 infections

**Figure 1.**
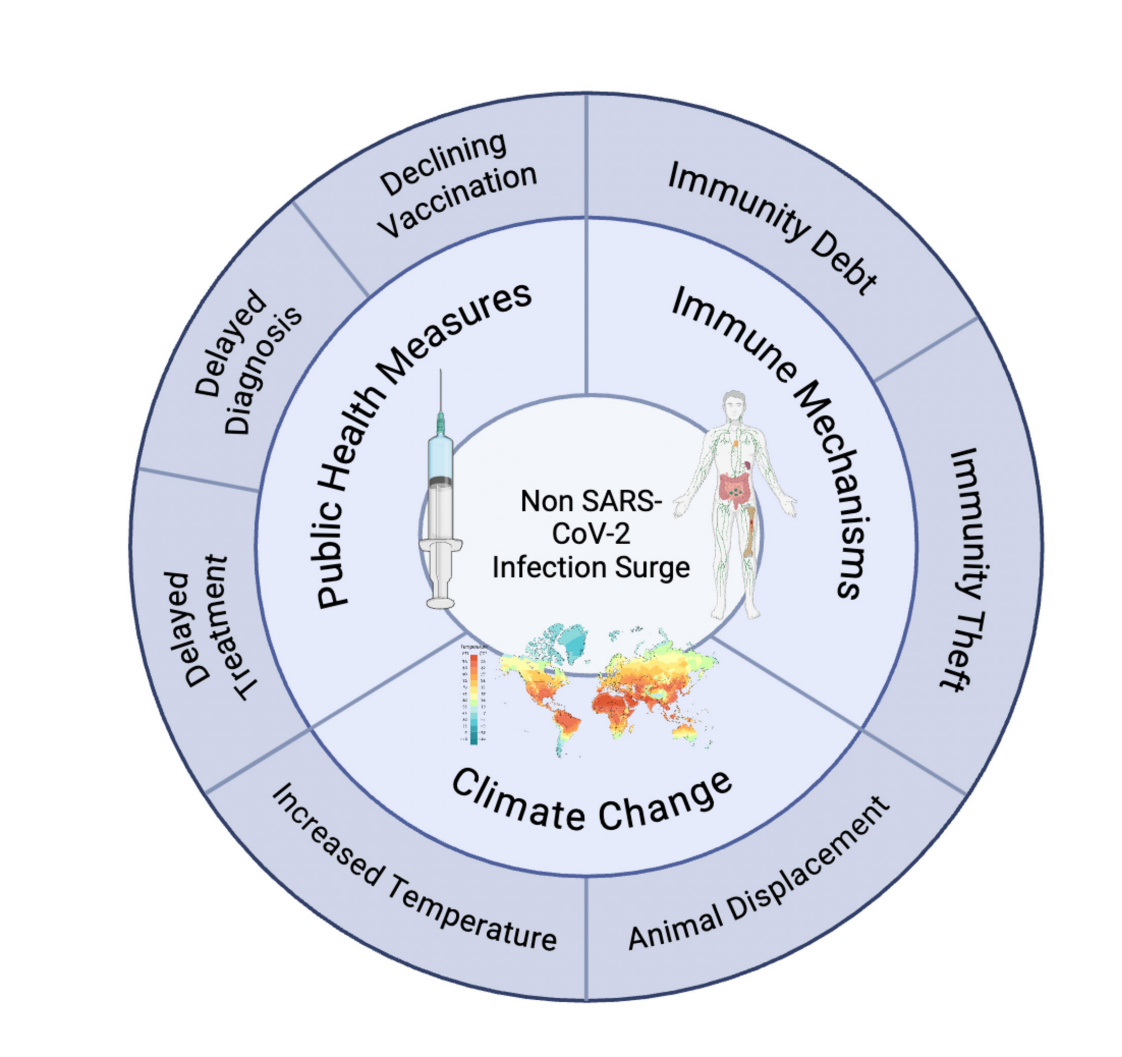
Potential mechanisms implicated in post-COVID-19 surge of non-SARS-CoV-2 infections. Some of the mechanisms and underlying causes that can explain the increased incidence of various non-SARS-CoV-2-driven infectious diseases following the COVID-19 pandemic are shown.

Although some forms of PASC were first reported in the very early stages of the pandemic (Callard and Perego, 2021), the changing epidemiology of infectious diseases throughout the world during and after the COVID-19 pandemic suggests that other pathogens may have contributed to the pathophysiology of PASC. For example, 44 countries have experienced a 10-fold increase in the incidence of at least 13 infectious diseases relative to the pre-pandemic baseline (http://www.airfinity.com/articles/global-surge-in-infectious-diseases-as-over-40-countries-report-outbreaks-10). Several non-mutually exclusive explanations have been proposed for this phenomenon (Figure 1). First, interruptions of public health measures may explain the emergence of some infections. For example, declining vaccine rates during the pandemic due to disrupted supply chains and limited immunization are likely to be major factors for increases in measles, polio, and whooping cough. Similarly, increases in tuberculosis cases may be related to the collapse of public health infrastructure, resulting in delayed diagnosis and treatment and failure of infection control measures (reviewed in Marco et al., 2024). Second, immune mechanisms may have contributed to a dramatic increase in respiratory viral infections, particularly in children (Rubin, 2024). One mechanism, popularized as ‘immunity theft’, describes a period of increased vulnerability to other infections following acute SARS-CoV-2 infection (Rubin, 2024). This mechanism is not only possible but probable, given that hyperinflammation of various microbial or viral origin is often followed by a period of immune suppression (Biswas and Lopez-Collazo, 2009; Libbey and Fujinami, 2002) and that PASC in children has been reported to give rise to prolonged periods of innate immune hyporesponsiveness (Khan et al., 2025). An alternative mechanism, known as ‘immunity debt’, which postulates a rebound of infections following a period of reduced exposure during lockdowns, widespread masking, and social distancing, has lost scientific support in favor of immune dysfunction hypotheses (Needle and Russell, 2023). Third, changes in temperature and rainfall patterns due to climate change may enable the spread of water- and mosquito-borne diseases such as cholera and dengue (Feinmann, 2024). A link between climate change and COVID-19 itself has been proposed (Ford et al., 2022), the implications of which are beyond the scope of the present article.

While causal links between the emergence of other viral and bacterial infections and the development of PASC have yet to be established, it is possible that the interactions of SARS-CoV-2 and other pathogens lead to PASC either directly or indirectly. For example (and as discussed below in more detail), SARS-CoV-2-induced immune dysregulation may increase susceptibility to other infections. Conversely, increased incidence of other infections may directly induce organ dysfunction or elicit immune dysregulation, leading to persistence of SARS-CoV-2 in the tissues. Regardless of the responsible mechanisms, further study of the interactions of SARS-CoV-2 and other pathogens, and their role in the development of PASC, is warranted.

## General considerations on co-infections and PASC

**Figure 2.**
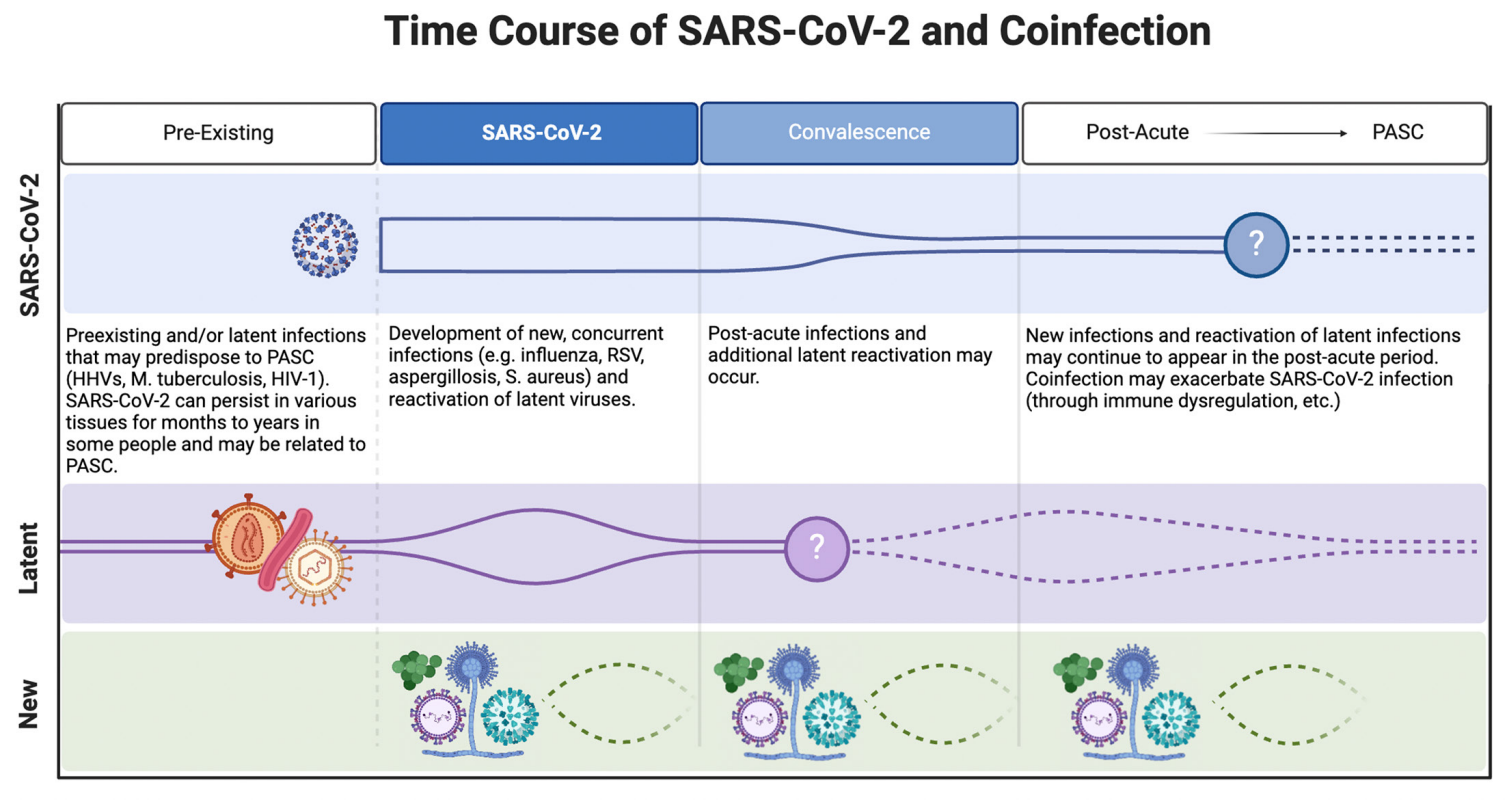
Potential temporal relationships between SARS-CoV-2 and co-infections. Various co-infections occurring at various times relative to the acute SARS-CoV-2 infection may predispose to post-acute sequelae of SARS-CoV-2 infection (PASC) or modulate the course of PASC.

Below, we briefly review current evidence on the role of SARS-CoV-2 persistence in tissues as a driver of PASC (Figure 2, top row). In the following sections, we discuss how, in the presence or absence of persistent SARS-CoV-2, other microorganisms may favor the development of PASC or exacerbate its severity. The discussion of causative relationships between PASC and co- or secondary infections requires considering multiple and intertwined elements. These include: (i) the type of co-infection (e.g. acute vs chronic, de novo infection vs reactivation of latent infection); (ii) mechanisms of pathogenesis, i.e., how infection with other agents may favor PASC development or worsen its severity and duration; (iii) temporal relationships, i.e., when co- or secondary infections have occurred relative to the acute SARS-CoV-2 infection, and (iv) the anatomical location of the infection.

## Does SARS-CoV-2 persist?

SARS-CoV-2 is transmitted through respiratory droplets and targets ciliated epithelial cells in the nasal cavity, trachea, and lungs by utilizing the cellular receptor angiotensin-converting enzyme 2 (ACE2) (Ahn et al., 2021; Jayaweera et al., 2020; Lamers and Haagmans, 2022; Jackson et al., 2022; Fraser et al., 2023). Infection limited to the upper respiratory tract is generally associated with milder disease outcomes, whereas dissemination to the lungs, in particular infection of the bronchi, bronchioles, and alveoli, can cause pneumonia, severe disease, acute respiratory distress syndrome, and death (Lamers and Haagmans, 2022). In addition, infected cells can be found in many other tissues throughout the body, particularly in vascular tissues. Studies in humans have described roles for type I and III interferons (IFNs), inflammatory cytokines (IL-6, TNF-α, IL-1β), innate immune cells (monocytes, pDCs, cDCs, natural killer cells), antibodies (IgG, sIgA, neutralizing antibodies), and adaptive immune cells (B cells, CD8^+^ and CD4^+^ T cells) in both pathogenesis and clearance of SARS-CoV-2 infection within the respiratory tract (Sievers et al., 2024; Harne et al., 2023).

The term *infection* can have a wide-ranging definition when referring to cells or tissues that harbor SARS-CoV-2 virus. This ambiguity often leads to varying interpretations of whether persistent SARS-CoV-2 infection is the cause of PASC symptoms. The definition of infection is rooted in the concept of tropism, which refers to the ability of a virus to infect a particular cell type or tissue. SARS-CoV-2 infection itself requires that a cell type expresses the ACE2 receptor, which can be ubiquitously expressed on cells within the respiratory tract and extrapulmonary tissues (Hamming et al., 2004; Ortiz et al., 2020). Historically, scientists relied on a rigorous definition of infection, which involved demonstrating the production of a virus in a cell type either by measurement of infectious virus (e.g. plaque assay, focus forming assay, outgrowth assay) or electron microscopy to visualize virions. These techniques are often difficult to perform or require specialized facilities/equipment. Alternatively, infection is defined in the literature by the presence of viral antigen, viral RNA using in situ hybridization or highly sensitive quantitative reverse transcription PCR (qRT-PCR), or viral sequencing. With these now routine, but less rigorous definitions, caution must be taken to avoid over-interpreting the impact that infection can have on PASC symptoms. For instance, viral antigen can persist for several weeks to months without any active virus replication in cells. Similarly, qRT-PCR or viral sequencing data can be from residual RNA in a particular cell type or tissue that has yet to be cleared following infection. In this section, we will look at findings within the respiratory tract in the context of acute and persistent SARS-CoV-2 infection.

To better understand SARS-CoV-2 persistence, it is important to consider the known cellular targets of SARS-CoV-2 during acute infection. Extensive studies using in vitro and in vivo (e.g. mice, hamsters, and nonhuman primates) infection models and specimens from infected humans have identified key cellular targets of SARS-CoV-2 infection. In the upper respiratory tract, SARS-CoV-2 infects the olfactory epithelium, mainly sustentacular cells, which can result in its rapid and gross desquamation (Khan et al., 2021; Verma et al., 2022). The resulting anosmia, defined by the loss of the senses of smell and taste, was described early in the pandemic and recognized as a profound neurological manifestation of COVID-19 (Tsukahara et al., 2023). A recent study suggests that a transition from the olfactory to the respiratory epithelium from the ancestral variants (e.g. WA1 and Delta) to the Omicron variants may account for the reduced incidence of anosmia in the Omicron era (Chen et al., 2024). Within the trachea, SARS-CoV-2 can infect epithelial cells of the mucosa, including goblet, ciliated, and basal cells. In the lungs, SARS-CoV-2 infects epithelial cells of the bronchi/bronchioles, including basal, ciliated, type II pneumocytes (ATII), and club cells (Hou et al., 2020). Other studies have identified macrophage subsets, including interstitial macrophages and alveolar macrophages, as potential targets of infection based on the presence of viral antigen or viral RNA (Huot et al., 2023; Wu et al., 2024). However, this observation may be a result of either phagocytosed infected cell debris or abortive infection, defined by a likely block in virus replication. The impact of SARS-CoV-2 infection of macrophages is not yet clear but may lead to dysregulated airway repair mechanisms (Wendisch et al., 2021). Macrophages have also been identified as potential targets of persisting SARS-CoV-2 (Huot et al., 2023).

The persistence of SARS-CoV-2 spike protein several months after a PCR-confirmed test has become a central tenet in the pathology of PASC. In a RECOVER-led multi-cohort study, SARS-CoV-2 spike can be detected in the blood for up to 14 months with around 50% of those experiencing persistent cardiopulmonary, musculoskeletal, and neurological symptoms also showing spike in the blood (Swank et al., 2024). It is also important to note that around 20% of asymptomatic individuals also showed circulating spike for up to 7 months post-recovery. It is not clear from this study whether a delay in viral clearance within the lungs or viral persistence in extrapulmonary tissues is the cause of persistent spike antigen in the blood. Autopsy studies have observed SARS-CoV-2 antigen and viral RNA in several extrapulmonary tissues, central nervous system, and the brain (Stein et al., 2022; Deinhardt-Emmer et al., 2021; Maffia-Bizzozero et al., 2023; Pesti et al., 2023). A longitudinal cohort study found that persistent inflammation characterized by type II interferon signaling and neutrophil activation was observed in individuals experiencing SARS-CoV-2 symptoms for more than 2 months (Talla et al., 2023).

## Types of co-infection

Various types of co-infection (i.e. those coincident with active SARS-CoV-2) may be implicated in the development or exacerbation of PASC. These include:

- acute infections by respiratory or other pathogens (e.g. influenza virus, respiratory syncytial virus [RSV], measles virus, adenovirus, *Staphylococcus aureus*, streptococci, *Hemophilus* spp., *Moraxella* spp., *Klebsiella pneumoniae, Acinetobacter, Pseudomonas, Aspergillus*).
- chronic infections (e.g. human immunodeficiency virus [HIV], cytomegalovirus [CMV], tuberculosis) and reactivation of latent infections (e.g. EBV, varicella zoster virus [VZV], or latent tuberculosis).

## Mechanisms of pathogenesis

Our in-depth understanding of the pathogenesis of many infectious diseases points to the biological plausibility of various direct pathophysiological mechanisms such as PASC drivers. For example, co-infections may predispose to PASC development by inducing altered organ function, immune dysregulation, autoimmunity, and organ damage (Box 1). Of note, noxious effects may be reciprocal, with pre-existing and concurrent co-infections worsening SARS-CoV-2 infection outcome and post-acute sequelae, and SARS-CoV-2 infection increasing the risk of superinfections during acute COVID-19 and post-acute secondary infections. While this is a burgeoning area of research, few studies have been able to make causal links between co-infections and PASC pathology. The following section is speculative, but we highlight how temporal and anatomical locations, as well as chronic infections, may influence the development of PASC.

Box 1. **How co-infections may induce post-acute sequelae of SARS-CoV-2 (PASC).**

- By presenting the incoming SARS-CoV-2 with a microenvironment that is already immunologically or functionally altered.
- By reactivating from latent reservoirs during acute or convalescent COVID-19.
- By taking advantage of perturbed immune responses during acute or early convalescent COVID-19.
- By triggering autoreactive adaptive immune responses via molecular mimicry or by reducing regulatory immune functions.
- By inducing immunosuppressive mechanisms (e.g. measles virus).
- By reducing mucosal (e.g. lung and/or gut) barrier functions.
- By altering the composition of microbiota.
- By potentiating noninfectious drivers of PASC through immune activation and systemic inflammation.

## Temporal relationships

Infection with any pathogen – may it be viruses, bacteria, and/or fungi – may precede, occur concurrently with (or soon after), or follow the onset of acute SARS-CoV-2 infection (Figure 2). Development of PASC may be induced or facilitated by pre-existing and/or latent infections, via dysregulated immune responses and/or dysfunctional organ systems. New infections and reactivation of latent infections that develop during (or soon after) acute SARS-CoV-2 infection may potentiate PASC by worsening histopathology and/or immune dysregulation. Furthermore, new or reactivated secondary infections may occur in the weeks or months following SARS-CoV-2 infection. In this case, it may be envisioned that SARS-CoV-2-induced immune dysregulation increases susceptibility to secondary infections or that, conversely, the effects of SARS-CoV-2 infection are exacerbated by immune dysregulation by ensuing infections, or both.

## Anatomical location: respiratory infections

**Figure 3.**
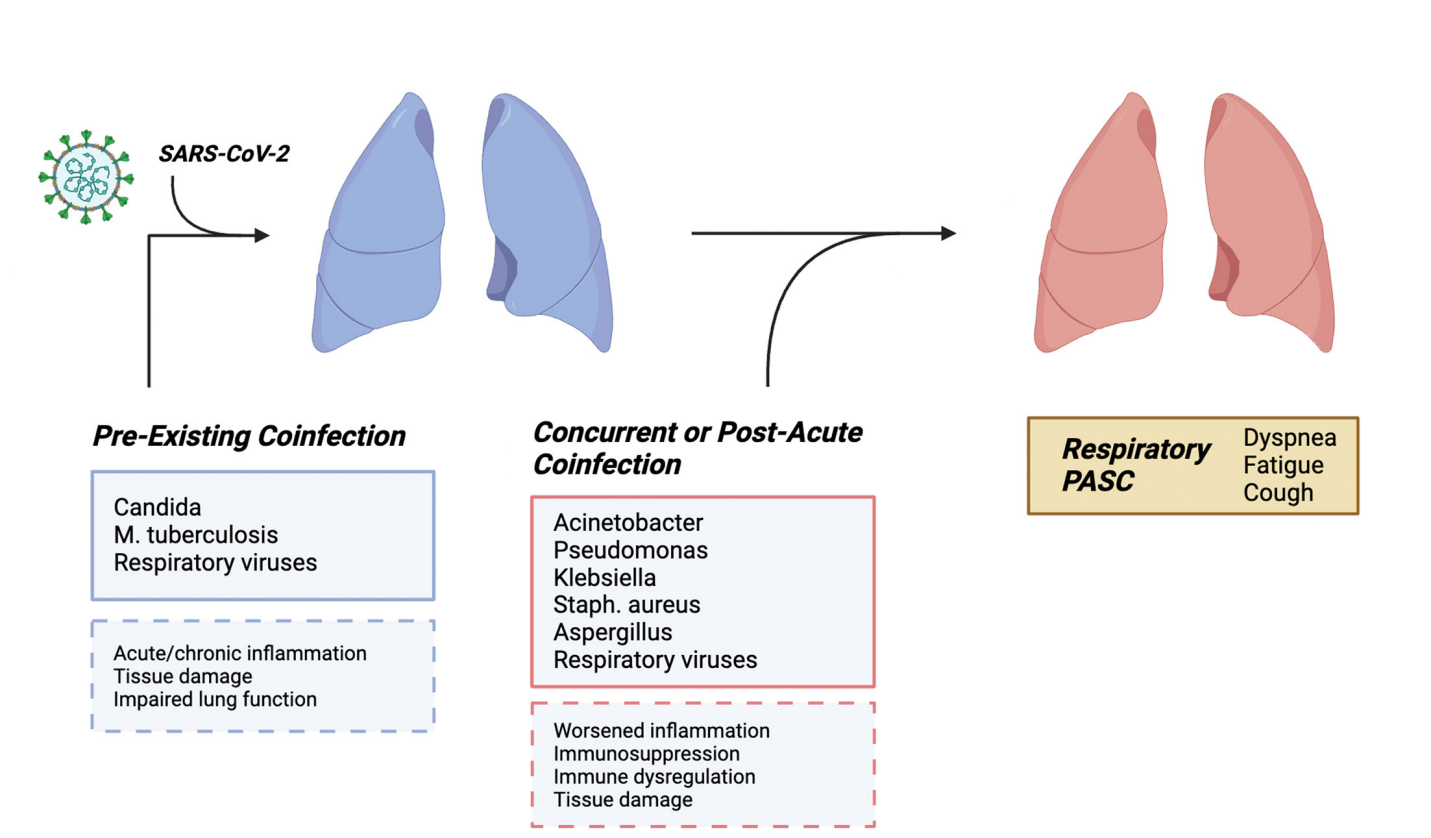
How co-infections may result in post-acute sequelae of SARS-CoV-2 (PASC) development in an organ system: the example of the lung. Pre-existing fungal, bacterial, and viral infections may result in acute and chronic inflammation, parenchymal damage, immune activation, and impaired lung function. For example, latent *Mycobacterium tuberculosis* infection may be reactivated during acute COVID-19, worsen COVID-19 clinical outcome, and/or lead to the development of PASC. Other pathogens such as those causing bacterial or fungal pneumonia during acute SARS-CoV-2 infection or early convalescence may also exacerbate lung tissue damage, cardiopulmonary function, systemic inflammation, and immune dysregulation, and favor development of PASC.

An additional set of considerations relates to anatomical location. As mentioned above, PASC may target various end organs such as the lungs, heart, and brain (Altmann et al., 2023). The lungs are a primary target organ during SARS-CoV-2 infection, and respiratory symptoms are important sequelae of PASC. It stands to reason, therefore, that respiratory infections due to other pathogens, including viruses, bacteria, and fungi, may modify risk factors for the development of PASC (Figure 3). For example, pre-existing acute or chronic inflammation, tissue damage, or impaired lung function may predate SARS-CoV-2 infection. Similarly, concurrent or post-acute co-infection with other pathogens may result in worsened inflammation, immunosuppression, immune dysregulation, or exacerbated tissue damage. In turn, these co-infections may then predispose to the development of respiratory PASC and associate dyspnea, fatigue, and cough. Below, we discuss some of the respiratory pathogens that may most conceivably contribute to PASC development.

### Respiratory viruses

The onset of the COVID-19 pandemic led to dramatic changes in the landscape of seasonal respiratory viral infections, due to social/physical distancing, lockdowns, dramatic decrease in attendance to restaurants, movie theatres, , shifting to at-home schooling, and the widespread use of masks in some geographic locations (reviewed in Chow et al., 2023). The role of seasonal and endemic respiratory viruses (e.g*.* influenza virus, RSV, rhinovirus, etc.) in the development or the clinical course of PASC remains unclear. Overall, rates of infection with enveloped respiratory viruses, such as influenza, RSV, common cold, human coronaviruses, and para-influenza viruses, were lower than usual during stricter pandemic non-pharmacological interventions (NPIs), e.g., masks, social distancing, reduced travel, school closings (Binns et al., 2022; Nasrullah et al., 2023). Resurgence has been identified following easing of NPIs. Infections with non-enveloped respiratory viruses, such as respiratory adenoviruses and especially rhinovirus, were less affected by NPIs during the pandemic (Chow et al., 2023). This may be associated with their greater environmental persistence, which might promote a more prevalent fomite-associated vs direct respiratory droplets and aerosol transmission. Acute or early convalescent SARS-CoV-2 infection may reduce the risk of acquiring a second viral infection through boosted innate immune responses (e.g*.* increased interferon production). Conversely, prior infection with a respiratory virus, as well as prior immunization against other agents (Netea et al., 2023), might also reduce the ability of SARS-CoV-2 to infect, likely due to increased innate immunity.

#### Influenza and RSV

Rates of influenza were quite low during the initial 2 years of the COVID-19 pandemic, likely due to NPI across the globe (Lu et al., 2022; Doroshenko et al., 2021). With the easing of COVID-19 restrictions, influenza re-emerged with an early surge in cases in the fall of 2022. Interestingly, hospital emergency visits showed an increase in RSV and influenza infections, peaking in December 2022 (Boehm et al., 2023; Rao et al., 2023), following an earlier peak of COVID-19 caused by the Omicron variant in August 2022. For example, emergency room visits for RSV dramatically increased during the fall of 2022, predominantly involving children aged 4 years and under (Boehm et al., 2023; Rao et al., 2023). This combined respiratory viral surge placed an elevated level of stress on pediatric healthcare systems. A major outstanding question is whether infection with influenza or RSV in subjects with pre-existing PASC would lead to PASC exacerbation. It is also unknown whether recent non-COVID-19 respiratory infections impact the course of subsequent SARS-CoV-2 infection or the development of PASC. Vaccination for influenza is a major intervention to reduce influenza burden, and combination influenza/COVID-19 vaccines are now available to simultaneously tackle both infections. However, the usefulness of these vaccines on PASC is yet to be evaluated.

#### Non-COVID-19 beta-coronaviruses

Other endemic coronaviruses such as alpha- and beta-coronaviruses are less pathogenic compared to SARS-CoVs. The human beta-coronaviruses OC43 and HKU-1 are more closely related to SARS-CoV-2 than are human alpha-coronaviruses 229E and NL63 (Cui et al., 2019). Human coronavirus OC43 and HKU-1 are responsible mostly for mild respiratory symptoms in the general population, although they can cause severe diseases in vulnerable individuals (Cui et al., 2019). There was early evidence of cross-immunity of SARS-CoV-2 with other human beta-coronaviruses that may have played a role in mediating severity of acute infection, thereby hypothetically reducing the risk of PASC. For instance, SARS-CoV-2 infection may boost memory B cells producing antibodies that bind to the most conserved S2 region and that are present in people with pre-existing immunity to seasonal human beta-coronaviruses (Aydillo et al., 2021). There is also evidence of cross-reactive T cell responses among human coronaviruses and SARS-CoV-2 (Mateus et al., 2020). Interestingly, people with PASC were found more likely to have SARS-CoV-2 antibody responses that cross-react with the less pathogenic OC43 strain (Herman et al., 2023; Spatola et al., 2023). However, it is not yet clear how cross-reactive antibody or T cell responses between OC43 and HKU-1 could lead to dysregulated immune responses that participate in the development of PASC.

#### Adenovirus

Infection with these viruses has an unclear role in PASC, but adenovirus infections have been recently associated with severe and fatal hepatitis outbreaks in children (Servellita et al., 2023). Many of these children had co-infection with SARS-CoV-2 and human herpesvirus (HHVs) (Ho et al., 2023), suggesting the possibility that these viruses may potentiate the pathogenicity of co-infecting viruses. However, no experimental evidence has demonstrated a link between adenovirus and the development of PASC. Future efforts to routinely conduct diagnostics for the most common viruses, including respiratory viruses, might improve our understanding of the impact of viral co-infections on health.

#### Rhinovirus

The circulation of these viruses was less affected by the COVID-19 pandemic compared to other respiratory viruses (Teo et al., 2022). It cannot be excluded that, as with other respiratory viruses, rhinovirus may enhance or reduce acute or post-acute effects of SARS-CoV-2 infection by driving inflammation or by promoting increased innate antiviral responses, respectively. However, given the co-circulation of rhinovirus and SARS-CoV-2, there is no direct evidence that rhinovirus infection is associated with PASC, and additional studies are warranted .

#### Measles virus

Co-infections with both measles virus and SARS-CoV-2 have been documented, though they appear to be rare (Nascimento et al., 2020). It is known that measles virus infections are associated with loss of B cell memory (Petrova et al., 2019; Mina et al., 2019). As some PASC symptoms appear to be associated with immune dysregulation resulting in reactivation of chronic infections, measles infection in PASC patients might further exacerbate PASC symptoms by decreasing memory B cells specific for chronic viruses. However, there is currently no evidence suggesting that co-infection necessarily leads to more severe disease or sequelae. In any case, measles vaccination is the best way to prevent this possibility.

### Respiratory bacteria

Bacterial infections occurring during acute COVID-19 (i.e. detected within the first 48 hr) are relatively rare. Although early reports suggested a high rate of bacterial co-infection at presentation (Langford et al., 2020), most studies report rates between 3% and 9% (Petty et al., 2022; Vaughn et al., 2021; Cong et al., 2022; Cheng et al., 2020; Karaba et al., 2021; Moreno-García et al., 2022). Of these, about half occur in the respiratory tract and are caused by common, community-associated pathogens, including *Staphylococcus aureus*, *Moraxella catarrhalis*, *Hemophilus influenzae*, *Streptococcus* spp., and *Escherichia coli* (Petty et al., 2022; Cheng et al., 2020; Calcagno et al., 2021). It remains difficult to discriminate benign colonization, which may occur in more than half of individuals (Kolenda et al., 2020; Sharifipour et al., 2020), from symptomatic bacterial respiratory co-infections, which have been reported in less than 5% of the population (Vaughn et al., 2021; Russell et al., 2021; Karami et al., 2021). Bloodstream infections comprise approximately the other half of acute co-infections and are most commonly caused by *S. aureus*, coagulase-negative staphylococci, *E. coli*, *Enterococcus faecalis*, *Acinetobacter* spp., and *Pseudomonas aeruginosa* (Petty et al., 2022; Vaughn et al., 2021; Karaba et al., 2021; Sepulveda et al., 2020; Khatri et al., 2021). Differences in case definitions, isolation methods, geographic variability, and time periods make interpretation of the myriads of studies difficult, but the consensus in the field is that bacterial co-infections during acute COVID-19 are relatively rare.

In contrast, secondary bacterial infections during or following treatment of COVID-19 are more common, with estimates ranging between 10% and 40% depending on the clinical setting (Petty et al., 2022; Cong et al., 2022; Cheng et al., 2020; Ripa et al., 2022; Ripa et al., 2021; Grasselli et al., 2021b; Hedberg et al., 2022; Murgia et al., 2023; Budhiraja et al., 2022). Not surprisingly, rates of secondary pneumonia (frequently ventilator-associated) and bacteremia (frequently catheter-associated) are more common in critically ill patients (Petty et al., 2022; Cong et al., 2022; Cheng et al., 2020; Ripa et al., 2022; Ripa et al., 2021; Hedberg et al., 2022; Murgia et al., 2023; Budhiraja et al., 2022; Grasselli et al., 2021a). The bacterial species most commonly isolated in these patients include *S. aureus*, *K. pneumoniae*, *Enterobacter* spp., *P. aeruginosa*, *Serratia* spp., *Acinetobacter baumannii,* and *E. coli* (Calcagno et al., 2021; Hedberg et al., 2022; Murgia et al., 2023; Buehler et al., 2021; Ceballos et al., 2022; Chen et al., 2023; Bartoszewicz et al., 2023), reflecting the healthcare-associated nature of these infections. In some locations, secondary infections with multidrug-resistant gram-negative bacteria have also been reported (Ripa et al., 2021; Budhiraja et al., 2022; Bork et al., 2021). Moreover, bacterial bloodstream infections were more common in hospitalized COVID-19 patients (3–4%) (Petty et al., 2022; Khatri et al., 2021; Ripa et al., 2021; Grasselli et al., 2021b; Murgia et al., 2023) than during mild acute infection, likely due to a combination of prolonged hospitalization and immunosuppressive therapy to treat COVID-19 (Khatri et al., 2021; Rothe et al., 2021). There are conflicting reports of whether secondary bacterial infection is more common among COVID-19 patients compared with those infected with other respiratory pathogens (Hedberg et al., 2022; Thelen et al., 2021; Youngs et al., 2020). It remains unclear whether SARS-CoV-2 itself specifically increased the susceptibility to bacterial superinfection, or the high rates reflect instead the severity of infection, use of ventilators, and prolonged hospital course, which are also known to be associated with increased risk of secondary infection.

While initial studies suggested that co-infections do not exacerbate COVID-19 symptoms (Cheng et al., 2020; Grasselli et al., 2021b), most reports demonstrate higher mortality, longer hospital length of stay, and longer duration of ventilatory support in patients with bacterial co-infection (Petty et al., 2022; Ripa et al., 2021; Grasselli et al., 2021b; Murgia et al., 2023; Budhiraja et al., 2022; Buehler et al., 2021). This raises the possibility that bacterial co-infection may predispose to PASC. For example, one would anticipate that longer duration of ventilatory support might increase long-term pulmonary inflammation and subsequent fibrosis, a proposed mechanism for PASC. Indeed, pulmonary infection following recovery from acute COVID-19 is common in survivors (Jakubec and Genzor, 2022) and could reflect either heightened susceptibility to secondary consequences of COVID-19 or a causative role for late-onset infections in mediating PASC. While there is no direct evidence at this time that bacterial infections contribute to the development of PASC, these studies together highlight the need for additional investigations. Specific questions that should be addressed include: (1) Does bacterial co-infection, either at presentation or developing during COVID-19, contribute to the development of PASC? (2) Is secondary bacterial infection a marker for PASC? (3) Can undiagnosed co-infections be found in patients with relatively mild COVID-19 disease who developed PASC? (4) Are secondary infections merely a marker for severe COVID-19 or do they affect the severity of COVID-19? (5) How can we discriminate severe COVID-19 and the postinfection course from post-ICU syndrome sequelae?

#### M. tuberculosis

Given the global burden of tuberculosis (TB) (over 10 million cases and 1.6 million deaths worldwide in 2021; http://www.who.int/publications/i/item/9789240061729) and the large reservoir of people latently infected with *M. tuberculosis* (estimated as one-quarter of the world population; Cohen et al., 2019), the question has arisen of whether pre-existing *M. tuberculosis* infections increase the risk of PASC or other severe manifestations of COVID-19 and, vice versa, whether infection with SARS-CoV-2 may trigger TB reactivation in latently infected individuals. Some evidence exists, mostly based on meta-analyses, that TB (with or without concurrent HIV infection) increases COVID-19 severity and mortality (Boulle et al., 2021; Tamuzi et al., 2020). A recent, multi-site register-based cohort study further supports a deleterious interaction between COVID-19 and TB, as co-infection was found associated with increased mortality, particularly in high-risk subjects, such as elderly males (Group, 2022), who are also highly susceptible to COVID-19 or TB alone (Zhou et al., 2020; Nhamoyebonde and Leslie, 2014).

The observed harmful interactions between these two infections may have a biological basis. For example, immunological measurements have shown that (1) HIV-induced lymphopenia in HIV-TB patients was associated with reduced anti-viral antibody levels and frequencies of SARS-CoV-2-specific CD4^+^ T cells; (2) active TB was associated with reduced polyfunctionality of SARS-CoV-2-specific CD4^+^ T cell responses; and (3) acute SARS-CoV-2 infection diminished the pool of *M. tuberculosis*-specific memory T cell responses (Riou et al., 2021), possibly due to SARS-CoV-2-induced lymphopenia (Shariq et al., 2022). Thus, concurrent *M. tuberculosis* and SARS-CoV-2 infection can reciprocally reduce the magnitude and possibly the effectiveness of the CD4^+^ T cell response to the co-infecting pathogen. Other biological mechanisms have been invoked, including the *M. tuberculosis*-induced upregulation of ACE2 (Shariq et al., 2022), which is a critical SARS-CoV-2 receptor on host cells (Hoffmann et al., 2020). When interpreting epidemiological data, however, it is important to consider that potential bias may be introduced by unmeasured confounding factors (Boulle et al., 2021), particularly because the populations concurrently exposed to these infections may be highly vulnerable due to socioeconomic and geographic factors (Duarte et al., 2021). Moreover, it is widely recognized that the COVID-19 pandemic has had a devastating impact on essential tuberculosis control worldwide (Dheda et al., 2022), resulting in increased tuberculosis morbidity and mortality in 2021, following decades of decline (http://www.who.int/publications/i/item/9789240061729).

Given the clinical spectrum of *M. tuberculosis* infection (Barry et al., 2009) and the abovementioned enormous burden of asymptomatic infection (latent *M. tuberculosis* infection [LTBI]), concerns exist that SARS-CoV-2 infection in patients with LTBI may increase the risk of progression to active TB (Group, 2022; Shariq et al., 2022). These concerns are justified, since SARS-CoV-2 infection may induce lymphopenia (Shariq et al., 2022) and, in particular, as mentioned above , reduce the pool of *M. tuberculosis*-specific CD4^+^ T cells (Riou et al., 2021), which are central to immune control of *M. tuberculosis* infection (Flynn and Chan, 2022). Moreover, the use of corticosteroids in COVID-19 treatment may increase the risk of TB reactivation in latently infected patients (Friedman and DeGeorge, 2022). Assessing the association between history of SARS-CoV-2 infection and TB reactivation – at a large scale, and beyond occasional case reports (Khayat et al., 2021; Leonso et al., 2022) – will prove challenging due to the sources of bias mentioned above and the need for large-scale prospective studies.

Whether and how TB might contribute to PASC is even less understood. TB may give rise to chronic lung abnormalities in subjects who have completed therapy, referred to as post-TB lung disease (PTLD) (Allwood et al., 2020). PTLD is poorly recognized and insufficiently studied, despite its relatively high prevalence (lung impairment following pulmonary TB is found in 18–87% of cases, depending on the setting; Allwood et al., 2020) and its association with reduced quality of life. PTLD is highly heterogeneous, as it may include airflow obstruction, restrictive ventilatory defects, and/or impairment in gas exchange, which are in turn associated with various lung immunopathological processes, such as tissue necrosis, cavitation, and aberrant tissue repair leading to fibrosis (Ravimohan et al., 2018). These processes are mediated mostly by proinflammatory and fibrogenic cytokines and chemokines, and tissue-degrading enzymes such as matrix metalloproteinases (Ravimohan et al., 2018). While still speculative, it is quite possible that the TB-induced chronic lung damage may not only increase the risk of severe acute COVID-19 but also favor additional chronic lung damage following SARS-CoV-2 infection. Indeed, precedents exist for respiratory infections, such as aspergillosis, to become chronic due to underlying lung damage by preceding infectious (TB, nontuberculous mycobacterioses) or noninfectious diseases (sarcoidosis, chronic obstructive pulmonary disease) (Kosmidis and Denning, 2015).

#### Non-tuberculous mycobacteria

Non-tuberculous mycobacteria (NTM) comprise approximately 200 bacterial saprophytic species that are found in the environment, particularly soil and water, and can cause opportunistic infections in the presence of host risk factors, such as old age, immunosuppression, and underlying chronic lung disease (in particular cystic fibrosis or bronchiectasis) (Johansen et al., 2020). Little is known about the association between NTM and COVID-19. Isolated cases of co-infection have been reported (e.g. Rodriguez et al., 2021; Masoumi et al., 2021), and a single-center study in Korea including almost 4000 subjects reported an excess of COVID-19 incidence in subjects treated for NTM (Kim et al., 2022) relative to the general population. However, a National COVID Collaborative Cohort (N3C) study including >6 million subjects failed to observe increased COVID-19 morbidity and mortality associated with pulmonary NTM disease (Figueroa Castro and Hersh, 2021). Even less is known about NTM infection and increased risk of PASC. However, it is plausible that lung damage resulting from severe COVID-19 might increase the risk of subsequent NTM infection.

### Respiratory fungi

Individuals with SARS-CoV-2 infection have increased susceptibility to invasive fungal infections caused by three major fungal pathogens: *Aspergillus*, *Zygomycetes*, and *Candida*. Since the first reported co-infection with *Aspergillus* in China in 2020, many cases of severe COVID-associated pulmonary aspergillosis have been described (Arastehfar et al., 2020b; Koehler et al., 2020; Prattes et al., 2022; Permpalung et al., 2022; Salmanton-García et al., 2021; Janssen et al., 2021). In 2021, COVID-19-associated mucormycosis emerged in India and was associated with high mortality rates (Ravani et al., 2021; Raut and Huy, 2021; Hoenigl et al., 2022). Invasive *Candida* infections in patients with COVID-19 are thought to represent opportunistic infections. Causative agents are *Candida albicans,* which is the most reported cause of candidiasis in critically ill patients (44% of candidemia cases in one US multicenter study), followed by *Candida auris* (Seagle et al., 2022; Arastehfar et al., 2020a; Jeffery-Smith et al., 2018). Risk factors associated with fungal co-infection include acute respiratory distress syndrome (ARDS) secondary to COVID-19, immunosuppressive therapies such as corticosteroids, and uncontrolled diabetes. Although frequently restricted to the lungs, fungal co-infection may often be disseminated with associated angioinvasion and severe tissue necrosis; such dissemination is associated with high mortality (up to 80%).

The potential mechanisms by which COVID-19 may predispose to severe fungal superinfection include epithelial damage, impaired ciliary clearance, pathologic immune activation and inflammation, and tissue necrosis. Conversely, fungal infection may exacerbate the severity of COVID-19 by inducing tissue necrosis and thrombosis, which may promote endothelial dysfunction and hypercoagulability, two cardinal features of severe COVID-19. As discussed above for viral and bacterial infections, the relationship between immune activation, endothelial dysfunction, and thrombosis associated with COVID-19 and fungal co-infection and the development of PASC remains unknown, indicating a need for future studies. Similarly, diagnostic challenges may have caused underestimation of the actual incidence and prevalence of fungal co-infections in patients with COVID-19. Future studies are needed to understand the pathophysiological mechanisms by which fungal co-infections may exacerbate the severity of COVID-19 and potentially contribute to the development of PASC.

## Anatomical location: extrapulmonary or systemic infections

**Figure 4.**
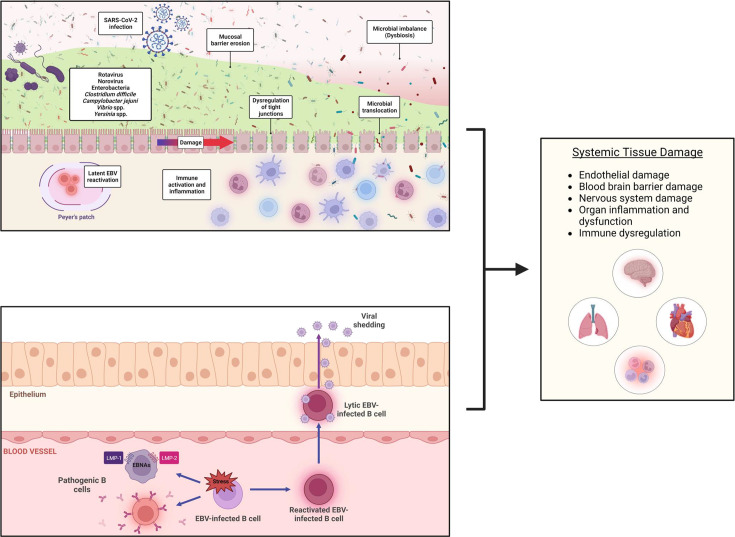
How co-infections may result in post-acute sequelae of SARS-CoV-2 (PASC) development systemically. The etiology of PASC is heterogeneous and involves multiple pathophysiological mechanisms. (**A**) shows how SARS-CoV-2 along with other viral and bacterial infections may compromise mucosal barriers (in this example, the intestinal lining) or change microbiome homeostasis leading to dysregulation of tight junctions, microbial translocation, immune activation, and systemic inflammation. (**B**) shows the potential impact of Epstein-Barr virus (EBV) reactivation during or following acute COVID-19. Viral shedding from lytic EBV-infected epithelial or B cells may lead to direct tissue damage, molecular mimicry, autoreactivity from host-cell expression of viral proteins, or pathogenic transformation of B cells that may traffic to various anatomical regions outside the areas of initial viral reactivation. All these mechanisms may lead to immune dysregulation and systemic tissue damage (**C**). LMP = latent membrane protein; EBNA = EBV nuclear antigen.

PASC may also be characterized by systemic symptomatology and/or the involvement of multiple organs. This is particularly relevant, since the ACE2 receptor, which is the cellular receptor by which SARS-CoV-2 enters cells, is widely distributed throughout the body. Systemic co-infections may play a causative/facilitating role in at least two different ways. One is by damaging mucosal barriers, with consequent microbial translocation and subepithelial immune activation, leading to systemic inflammation, endothelial damage, and compromised organ systems. The example of intestinal co-infections (or changes in gut microbiota) and gut lining damage as PASC drivers is depicted in Figure 4A, but systemic disease caused by bacterial and angioinvasive fungal infections (discussed above) could drive similar changes.

Another mechanism of pathogenesis of systemic PASC relates to the reactivation of latent viruses, such as EBV. It can be envisioned that SARS-CoV-2 infection may facilitate EBV reactivation in the Peyer’s patches in the gut (Figure 4A) or in lymphoid tissue elsewhere (Figure 4B), resulting in either viral shedding and direct tissue damage, systemic damage secondary to EBV-induced immune dysregulation or pathogenic transformation of B cells that may traffic to anatomical regions outside the areas of initial viral reactivation (Figure 4B). Thus, co-infections may be drivers of PASC, either at the site of infection or systemically, and induce a wide range of clinical phenotypes.

## Chronic (pre-existing) viral pathogens

### Epstein-Barr virus

Latent EBV is an HHV harbored by 95% of the world population usually defined by the presence of detectable, long-lived EBV viral capsid antigen IgG levels (Dunmire et al., 2018; Damania et al., 2022). EBV can infect B cells and epithelial cells (in addition to many other cell types) and fluctuate between latent and lytic infection (Damania et al., 2022). As a result, EBV can reactivate in the setting of various physiological stressors or dysregulated immune responses. In some cases, reactivated EBV in tissues may not manifest with detectable circulating DNA in blood (Guo et al., 2019; Middeldorp, 2015). While reactivation of EBV can lead to end-organ damage, most commonly in immunocompromised hosts, EBV infection can also lead to various malignancies such as Burkitt lymphoma, gastric carcinoma, Hodgkin lymphoma, nasopharyngeal carcinoma, B cell lymphomas, and posttransplant lymphoproliferative disease in which EBV viral gene expression has been detected (e.g*.* EBER, BART, EBNA, LMP) (Damania et al., 2022). Recent data suggest that EBV infection may drive multiple sclerosis (MS) (Bjornevik et al., 2022), perhaps due to aberrant autoreactive immune responses (Aloisi and Salvetti, 2022). EBV nuclear antigens 1 and 2 (EBNA1 and 2) are expressed during the latent and lytic phases of infection and have been demonstrated to exhibit molecular mimicry with human proteins involved in various autoimmune conditions (e.g. rheumatoid arthritis, systemic lupus erythematosus, type 1 diabetes, celiac disease, and MS) (Houen and Trier, 2020; Harley et al., 2018).

Whereas the precise causative relationship between EBV infection or reactivation and various rheumatological diseases is lacking, associations between EBV reactivation during acute SARS-CoV-2 infection and the development of various PASC symptom clusters have been reported. For example, a study published early during the COVID-19 pandemic identified EBV early antigen-diffuse (EA-D) IgG positivity, a marker that may indicate recent viral activity or reactivation, in two-thirds of individuals experiencing PASC; in particular, EBV EA-D IgG levels were higher in those with more PASC symptoms (Gold et al., 2021). A subsequent study found that detectable EBV DNA during acute SARS-CoV-2 infection predicted the presence of symptoms at 30–60 days post-COVID-19 (Su et al., 2022), although the long-term impact on persistent symptoms was not studied. Another recent study of 280 participants showed that PASC symptoms such as fatigue and neurocognitive dysfunction 4 months following acute COVID-19 were independently associated with serological evidence of recent EBV reactivation (EBV EA-D positivity) or high EBNA1 IgG levels, but not with ongoing circulating EBV DNA (Peluso et al., 2023). EBV reactivation was also independently associated with fatigue, a hallmark feature of acute EBV infection/mononucleosis that can persist for weeks to months (Peluso et al., 2023). Another study showed that, in addition to EBV EA-D antibody responses, seropositivity to EBV envelope glycoproteins gp42 and gp350, which are essential for EBV lytic infection of B cells, was higher in participants with long COVID (Klein et al., 2023), also suggesting recent viral activity associated with the development of PASC (Karsten et al., 2022).

It is also of interest that oligoclonal bands, which represent immunoglobulins that are classically associated with MS, have been identified in the cerebrospinal fluid in approximately 70% of individuals with PASC compared with none in controls without PASC (Apple et al., 2022). Notably, of patients with MS, ~40% had oligoclonal bands in the cerebrospinal fluid that are reactive to HHVs, including human herpesvirus 6 (HHV-6), and EBV, compared to none in patients with other inflammatory neurological disorders (Virtanen et al., 2014). Additionally, these herpes-reactive oligoclonal bands in MS patients were also associated with the presence of viral DNA in the cerebrospinal fluid (Ravani et al., 2021). Therefore, it is possible that EBV reactivation in B cell-rich areas, such as lymph node follicles or bone marrow, may lead to autoreactive antibody responses. Indeed, EBV-infected, pathogenic B cells have been shown to migrate to the brain in MS patients (Damania et al., 2022). Further investigation is urgently needed to determine whether a similar autoimmune process may be involved in the development of PASC neuronal symptoms.

### Cytomegalovirus

CMV is another HHV that infects and persists in greater than 80% of the global population (Zuhair et al., 2019). Some groups, such as those living with HIV, exhibit a much higher prevalence of IgG positivity (>95%). Like other HHVs, CMV persists indefinitely in a predominantly quiescent state and can reactivate during physiological stress and dysfunctional immunity, leading to detectable circulating DNA and end-organ damage. CMV reactivation is relatively common in critically ill patients requiring intensive care, and treatment of CMV in these patients may reduce time on mechanical ventilation and improve oxygenation (Limaye et al., 2017). COVID-19 patients infected earlier during the pandemic who were CMV IgG+ experienced higher rates of hospitalization and overall morbidity during acute infection (Alanio et al., 2022; Weber et al., 2022). CMV can reactivate in anatomical locations similar to SARS-CoV-2, including the lungs and endovasculature, and may play a role in exacerbating the severity of COVID-19 progression.

The impact of underlying CMV infection (i.e. IgG positivity) was independently associated with a lower odds ratio of developing neurocognitive PASC, an association that was not observed with reports of fatigue (Peluso et al., 2023). Thus, CMV clearly plays a multifactorial role in modulating acute COVID-19 and PASC. One hypothesis is that CMV-seropositive individuals may mount more robust and durable adaptive immune responses to SARS-CoV-2 over time, perhaps due to higher basal levels of IFNγ (Barton et al., 2007). In support of this idea, CMV seropositivity in younger adults has been linked with more robust adaptive immune responses to influenza vaccination (Furman et al., 2015). Other studies have linked CMV infection to immunosenescent phenotypes (Nicoli et al., 2022). Furthermore, CMV-induced immunoregulatory pathways may reduce local inflammation or decrease autoantibody formation. While speculative, these mechanisms need further study.

### Other HHVs

HHV-1, HHV-6, and HHV-7 can reactivate in the setting of acute SARS-CoV-2 infection (Xu et al., 2020; Drago et al., 2021), but there is a paucity of data on reactivation or underlying seropositivity of other HHVs as potential drivers of PASC. However, given the complex interactions between EBV, CMV, and PASC, tissue and blood studies should include more detailed investigation into the potential role of these chronic viral infections in modulating PASC. Indeed, VZV glycoprotein E was found increased in PASC patients (Klein et al., 2023), providing further evidence that other HHVs may play a role in PASC pathogenesis.

### HIV-1

Emerging data suggest that people with HIV-1, especially those with underlying HIV-1-associated immune dysfunction, are more susceptible to acute SARS-CoV-2 infection or have worse clinical outcomes (Augello et al., 2023). Importantly, chronic HIV infection leads to detrimental chronic immune activation and inflammation (e.g. elevated IL-6, scCD14, sCD163, C-reactive protein [CRP], etc.), even after decades of suppressive antiretroviral therapy (ART), and people with HIV continue to have elevated risks of various cardiopulmonary, metabolic, and oncological diseases over time due to ongoing persistent inflammation (Borges et al., 2013; Sereti et al., 2017; Nordell et al., 2014; Tenorio et al., 2014; Kuller et al., 2008). Similar markers of aberrant immune activation and inflammation have been observed in the setting of various manifestations of PASC (e.g. IL-6, CRP) (Peluso et al., 2021b; Durstenfeld et al., 2022; Peluso et al., 2022d). Thus, the question arises whether pre-existing HIV infection can potentiate PASC (Augello et al., 2023). In at least some HIV-infected or otherwise immunodeficient individuals, inability to clear SARS-CoV-2 may lead to long-lasting, persistent infection, which is characterized by a much greater level of evolution and immune escape. It has been speculated that such coinfected individuals may be the source of the sudden appearance of new SARS variants, such as the Omicron variant in southern Africa in late 2021 (Dhama et al., 2023).

While the association between HIV and SARS-CoV-2 is understudied, several studies suggest that people with HIV, many on suppressive ART, have a 1.4- to 4-fold increase in PASC risk (Peluso et al., 2022e). IL-6 levels were also observed to be higher in PASC participants with HIV relative to those without HIV (Peluso et al., 2022e). Another investigation showed that pre-existing HIV infection was independently associated with higher odds (>2) of developing neurocognitive symptoms when controlled for demographic factors, initial disease severity, other underlying medical conditions, and CMV and EBV serological status (Peluso et al., 2023).

It is plausible that COVID-19 is essentially an insult added to an existing ‘injury’, exacerbating an already dysregulated and inflammatory environment, including increased gut microbial translocation and disruption of mucosal barriers observed in both HIV-1 and SARS-CoV-2 infections (Giron et al., 2022; Sheth et al., 2008; Haase, 2005; Schuetz et al., 2014). An increased frequency of HIV ‘blips’ or low-level, transient detectable HIV RNA copies in the blood has been reported in people with HIV on ART months following initial COVID-19 (Peluso et al., 2021a), suggesting that SARS-CoV-2 infection may also exacerbate smoldering, persistent HIV-1 activity, leading to a synergistic spiral of virus-induced inflammation.

### Hepatitis B and C viruses

Hepatitis C virus (HCV) infects up to 2% of the global population despite the availability in well-resourced settings of direct antivirals with high efficacy. Data are sparse, but some studies have suggested that people with HCV may have higher rates of hospitalization for COVID-19 and higher degrees of liver inflammation/damage, although ICU admission and mortality were not affected (Butt et al., 2021). Experimental data have also emerged that HCV may increase ACE2 expression on hepatocytes, potentially facilitating SARS-CoV-2 entry in liver tissue (Domovitz et al., 2022). However, the evidence of SARS-CoV-2 infection of the liver in humans is limited, and further tissue-based studies are warranted. Acute SARS-CoV-2 infection can also lead to HCV reactivation, but there is a paucity of data suggesting that HCV predisposes or plays a direct pathogenic role in the development of PASC. Reactivation of chronic hepatitis B virus (HBV) infection during acute COVID-19 is low, and individuals with cirrhosis may be at higher risk of severe disease (Marjot et al., 2021). As with HCV, however, there is no evidence supporting chronic HBV infection or reactivation with PASC.

## Non-respiratory bacterial infection

### Lyme disease

Concerns exist regarding *Borrelia burgdorferi* infection potentially predisposing patients to more severe symptoms of COVID-19, especially given the propensity for many individuals to develop postinfectious syndromes following acute borreliosis (Marques, 2022). The term chronic Lyme disease is used to describe distinct clinical manifestations of late Lyme disease, including progressive encephalomyelitis, chronic polyneuropathy, and chronic fatigue syndrome (CFS) (Bai and Richardson, 2023). The latter has been used frequently to refer to patients with chronic subjective complaints, such as fatigue, musculoskeletal pain, and cognitive dysfunction without a documented history of Lyme disease or evidence of an active infection with *B. burgdorferi* (Lim and Torpy, 2000). However, in some cases, evidence for infection based on serology or clinical presentations of Lyme has been documented (Bai and Richardson, 2023). In addition to serology, diagnosis of CFS associated with chronic Lyme disease is based on two assumptions: first, that chronic Lyme disease is caused by persistent infection with *B. burgdorferi*; and second, that this chronic infection form is unresponsive to conventional antimicrobial treatment.

Fatigue is a frequently documented component of PASC and persistent Lyme disease, as well as other viral infections such as EBV and influenza (Thaweethai et al., 2023; Wang et al., 2021; McCall et al., 2008). Interestingly, the presence of fatigue is consistent with later stages of these infectious conditions that share symptoms with noninfectious neurodegenerative states such as post‐traumatic brain injury and post-stroke (Thapa Magar et al., 2022; Mollayeva et al., 2014). In addition to fatigue, chronic Lyme disease and PASC are characterized by persistent or chronic neurological manifestations, including cognitive dysfunction, suggesting similar pathophysiological mechanisms (Marques, 2022; Thaweethai et al., 2023).

A recent study that combined serological and clinical data found that Borrelia-specific IgG antibodies correlated with COVID-19 severity (Szewczyk-Dąbrowska et al., 2022), with 31 out of 31 hospitalized COVID-19 patients, 19 out of 28 mild/asymptomatic, and only 8 out of 28 healthy controls positive for Borrelia-specific IgGs. A lower positivity was reported for Borrelia-specific IgM. While the authors evaluated cross-reactive antibodies against other bacterial and spirochetal species, such as *Anaplasma* and *Treponem*a, respectively, there remains a concern about the specificity of IgG antibodies against *Borrelia*. Serological diagnosis of Lyme disease, as well as tick-borne diseases, is challenging due to antibody cross-reactivity among several tick-borne pathogens, which causes false-positive results. Thus, serology-based diagnosis of co-infection with Borrelia and SARS-CoV-2 is questionable and requires further confirmation by other immunoassays or molecular nucleic acid amplification testing (van Gorkom et al., 2017; Lantos et al., 2015; Webber et al., 2019). Further studies combining multiple diagnostic approaches and larger cohorts of patients are needed to confirm these intriguing findings.

The mechanisms by which neurophysiological changes occur following recovery from acute infections with *Borrelia* or SARS-CoV-2 and the disappearance of acute systemic cytokine storm remain elusive. Murine studies demonstrated that systemic injection of lipopolysaccharide, a pathogen-associated molecular pattern (PAMP) constitutively expressed in Gram-negative bacteria including *Borrelia*, is followed by prolonged systemic TNF-α production and persistence in the cerebrospinal fluid (Qin et al., 2007). Whether similar PAMPs exist in SARS-CoV-2 and persist in the CSF following resolution of acute infection remains unknown. Nevertheless, access of these PAMPS to the CNS through the blood-brain barrier can activate microglia (resident macrophage-like cells) via TLR4, leading to local production of TNF and other inflammatory cytokines (Biswas, 2023). Understanding the shared pathophysiological mechanisms of chronic Lyme disease and PASC might have therapeutic and diagnostic implications.

## Animal models of PASC

Animal models, including mice, hamsters, and nonhuman primates (NHPs), have been used to study the pathogenesis, immunity, transmission, and protection against SARS-CoV-2 infection. These models have been essential for preclinical development and testing of countermeasures, which include vaccines, monoclonal antibodies, and direct-acting antivirals (Boras et al., 2021; Corbett et al., 2020; Guebre-Xabier et al., 2020; Hansen et al., 2020; He et al., 2021; Pruijssers et al., 2020; Tian et al., 2021; van Doremalen et al., 2020; Vogel et al., 2021).

Mice, hamsters, and NHPs have been used to define the contributions of the innate immune response, including type I IFNs, pro-inflammatory and anti-inflammatory cytokines (IL-6, TNF-α, IL-1β, IL-10, IL-2), and innate cell-mediated responses (macrophages, monocytes, neutrophils, eosinophils, natural killer cells), and the adaptive immune response (humoral, B cells, and T cells) in modulating protection against SARS-CoV-2 (Case et al., 2025). These animal models are now being leveraged to replicate facets of long COVID and serve as platforms for mechanistic studies and therapeutic development. Several review articles have been published describing animal models of gastrointestinal, neurological, and cardiovascular PASC and will not be discussed in this review (Vanderheiden and Diamond, 2025; Chung et al., 2025; Dai et al., 2025). We highlight how animal models are being used to study pulmonary sequelae and viral persistence.

Pulmonary sequelae, such as lung fibrosis and chronic inflammation, have been prominently observed during the recovery phase following SARS-CoV-2 infection of mice. For instance, infection of standard laboratory strains with a mouse-adapted SARS-CoV-2 strain (MA10) has been shown to cause chronic pulmonary epithelial and immune cell dysfunction with fibrosis, as well as sustained lung inflammation, injury, and airway wall thickening (Dinnon et al., 2022). Sustained neutrophil activation and extracellular trap formation (Giannakopoulos et al., 2024), complement (Perico et al., 2023), dysregulated macrophage-CD8^+^ T cell signaling axis (Narasimhan et al., 2024), and fibroblast-intrinsic CD147 (Wu et al., 2022) have been shown to contribute to SARS-CoV-2-induced progressive pulmonary fibrosis. Further, therapeutics including antivirals and monoclonal antibodies have been shown to reduce lung fibrosis and disease severity (Dinnon et al., 2022; Wu et al., 2022; Kolloli et al., 2023; Zhang et al., 2024).

Persistent SARS-CoV-2 reservoirs that remain hidden in various tissues have been postulated to drive chronic inflammation and symptoms long after acute infection. One of the challenges with animal models is that SARS-CoV-2 is rapidly cleared following acute infection, which has made it difficult to study viral persistence. CD4^+^ and CD8^+^ T cells have been shown to be recruited to the upper and lower respiratory tract following SARS-CoV-2 infection in mice (Kar et al., 2024). Antibody-based depletion of T cells resulted in persistent infectious virus in the upper respiratory tract, but not lungs, for more than a month. This finding demonstrates that T cells are required for preventing viral persistence in the respiratory tract. Treatment with antivirals cleared viral persistence in the upper respiratory tract (Lieber et al., 2024). In a reductionist model, intravenous delivery of the SARS-CoV-2 spike protein led to neuroinflammation within the skull-meninges-brain axis and induced anxiety-like behavior in mice (Rong et al., 2024).

Animal models should continue to be used to develop deeper mechanistic insight to the pathophysiology of PASC. There are many factors that should be considered, including age, sex, host genetics, immunological status (e.g. pre-existing vaccine or infection-induced immunity), metabolic state, sequential and/or co-infections with other heterologous pathogens, obesity, and diabetes. We should attempt to use models that appropriately recapitulate acute infection, pathogenesis, immune responses, and recovery observed in humans.

## Summary and implications for future research and clinical care

Here, we reviewed data regarding the interactions of pathogenic microorganisms with SARS-CoV-2 infection and the possibility that co-infections are associated with the development of PASC. Co-infections important to PASC can be pre-existing, reactivated during or after acute infection, or infect de novo along a wide time range. The relationship between SARS-CoV-2 and other co-infecting microorganisms may be multifaceted. On one hand, SARS-CoV-2 infection might exacerbate immunological dysfunctions caused by previous infections with other pathogens. On the other hand, immune dysregulation and tissue damage during SARS-CoV-2 infection might trigger reactivation of latent infections or predispose to severe disease from the subsequent encounter with other pathogens. In addition, co-infections might trigger severe manifestations and sequelae of SARS-CoV-2 infection that may not occur in the absence of co-infection. Moreover, various co-infections might have different consequences for PASC manifestations. Depending on the pathogen, the time between infections, and the presence of other predisposing host and environmental factors, co-infection-related effects may be organ-specific and/or have systemic implications, giving rise to a wide spectrum of sequelae of SARS-CoV-2 infection and diverse PASC symptoms. Given the multitude of clinical phenotypes and potential mechanistic drivers, small epidemiological and tissue or blood-based mechanistic studies are insufficient to demonstrate the causal roles of any given co-infection in PASC development. As a result, these studies should be complemented with large longitudinal studies, such as the RECOVER initiative, which have the potential to assess the association of various co-infections with PASC or specific PASC phenotypes across a wide cross-section of the population. A better understanding of pathogen-specific associations of co-infections with PASC will be critical in the development of appropriate diagnosis of long COVID patients. This, in turn, will help find PASC therapies tailored according to the specific underlying mechanism.

## Limitations

Due to the large heterogeneity of PASC phenotypes and the various co-variates that might drive them, including co-morbidities, genetic predisposition, previous exposure to various agents, and, as discussed in this manuscript, co-infections, we propose a framework to best conduct PASC research, which allows for informed comparisons among studies, so that advances can be achieved leading to specific therapies for the affected patients (Box 2). This framework involves the participation of clinicians, microbiologists, immunologists, pathologists, statisticians, bioinformaticians, machine learning and AI experts, patient advocates, regulators, and funders to ensure not only that samples are collected from large longitudinal studies, such as the RECOVER initiative, but also that the appropriate information is collected to address the multiple variables that can drive PASC symptoms, and that samples are analyzed in a comprehensive and comparative way to best establish correlates of disease and to generate hypotheses to further investigate possible therapies. With this framework, clinical trials targeting specific mechanisms identified in subsets of PASC patients, related or not related to co-infections, are likely to be more informative than clinical trials in PASC patients without a clear hypothesis on the underlying causes of their symptoms. In this respect, the development of animal models of PASC that investigate the impact of co-infections will also be of significant help for the discovery of preventive measures and therapies to mitigate the sequelae associated with SARS-CoV-2 infection. The implementation of adequate clinical strategies based on a better understanding of the factors associated with PASC, including the impact of co-infections, will pave the way for finding treatments that mitigate the development of PASC.

Box 2. **A framework to study the role of co-infections in post-acute sequelae of SARS-CoV-2 (PASC).**

- Establish a network of investigators, clinicians, patient advocates, regulators, and funders to continue large longitudinal studies that generate samples and clinical information from PASC patients, including those ascertaining the presence of co-infections.
- Analyze samples from large cohorts in ways, including machine learning and AI, that allow for correlations between data generated and clinical information.
- Generate hypotheses on drivers of PASC symptoms.
- Establish animal models, ex vivo systems, and human studies to investigate hypotheses and test therapies.
- Conduct informed clinical trials based on the inhibition of the mechanisms found to be involved in PASC phenotypes, including those driven by co-infections.
